# Transcriptome profiling of maternal stress‐induced wing dimorphism in pea aphids

**DOI:** 10.1002/ece3.5692

**Published:** 2019-10-02

**Authors:** Lin Hu, Wanying Gui, Bing Chen, Li Chen

**Affiliations:** ^1^ State Key Laboratory of Integrated Management of Pest Insects and Rodents Institute of Zoology Chinese Academy of Sciences Beijing China; ^2^ Key Laboratory of Beibu Gulf Environment Change and Resources Utilization of Ministry of Education Nanning Normal University Nanning China; ^3^ University of Chinese Academy of Science Beijing China; ^4^ College of Life Science Hebei University Baoding China

**Keywords:** alarm pheromone, crowding, maternal stress, pea aphid, predation, transcriptome profiling, wing dimorphism

## Abstract

Wing dimorphism, that is, wingless and winged forms, can be induced by maternal stress signals and is an adaptive response of aphids to environmental changes. Here, we investigated the ecological and molecular effects of three kinds of stress, namely crowding, predation, and aphid alarm pheromone, on wing dimorphism. These three stressors induced high proportion of up to 60% of winged morphs in offspring. Transcriptome analysis of stress‐treated female aphids revealed different changes in maternal gene expression induced by the three stressors. Crowding elicited widespread changes in the expression of genes involved in nutrient accumulation and energy mobilization. Distinct from crowding, predation caused dramatic expression changes in cuticle protein (CP) genes. Twenty‐three CP genes that belong to CP RR2 subfamily and are highly expressed in legs and embryos were greatly repressed by the presence of ladybird. By contrast, application of alarm pheromone, *E*‐β‐farnesene, caused slight changes in gene expression. The three factors shared a responsive gene, cuticle protein 43. This study reveals the adaptive response of aphids to environmental stresses and provides a rich resource on genome‐wide expression genes for exploring molecular mechanisms of ecological adaptation in aphids.

**OPEN RESEARCH BADGES:**

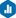

This article has earned an Open Data Badge for making publicly available the digitally‐shareable data necessary to reproduce the reported results. The data is available at https://doi.org/10.5061/dryad.55b2b15.

## Graphical abstract

We investigated the ecological and molecular effects of three kinds of maternal environment stress, namely crowding, predation, and aphid alarm pheromone, on offspring wing dimorphism in pea aphid. This study reveals the adaptive response of aphid to environmental stresses and provides a rich resource on genome‐wide expression genes for exploring molecular mechanisms of ecological adaptation in aphids.

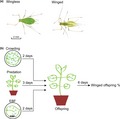

## 1 INTRODUCTION

Phenotype plasticity is a smart adaptive strategy universally acquired by many species in long‐term evolutionary processes. In a changing environment, organisms possessing high plasticity in their behavior, morphology, and physiology would exhibit great fitness in survival and reproduction (Chevin, Lande, & Mace, 2010; Simpson, Sword, & Lo, 2011). Aphids (Hemiptera: Aphididae), such as the pea aphid (*Acyrthosiphon pisum*), are the representative species that survives an adverse and fluctuating environment through exhibiting such strong phenotypic plasticity. In warm spring and summer, the parthenogenetic wingless females can produce large numbers of wingless offspring to rapidly increase their population. Much of the aphids' energy is devoted to reproduction (Dixon & Howard, 1986; Zhang, Wu, Wyckhuys, & Heimpel, 2009). By contrast, wingless females produce winged offspring to migrate to new well‐nourished plants when their host plant deteriorates and turn to sexual reproduction in autumn. The winged morphs input more energy on the development of wings and flight muscle rather than on reproduction (Brisson, Davis, & Stern, 2007). Therefore, the winged morphs present a smaller body size and less oviposition than the wingless morphs (Mackay & Wellington, 1975; Xu, Liu, Zhang, & Wu, 2011). The sexual female aphids produce eggs for overwintering through female and male copulation (Shingleton, Sisk, & Stern, 2003). Therefore, the interconversion between wing dimorphism and reproductive dimorphism can confer a selective advantage or disadvantage depending on environmental conditions. The various alternative phenotypes adopted by aphids to cope with the stressful environment make it an excellent model in studying adaptive dimorphism.

Abiotic and biotic environmental factors determine wing dimorphism in parthenogenetic female aphids. As a biotic factor, food source plays an important role in transgenerational wing dimorphism in aphids. Wingless females feeding on deteriorating plant sources promote production of winged offspring in *Aphis craccivora* and *A. pisum* (Johnson, 1966; Sutherland, 1969a, 1969b). Supplying wingless aphids with a continuous flow of artificial diet with poor nutrition can increase the percentage of winged offspring in *Myzus persicae* (Harrewijn, 1976). In addition to nutrient substances, defensive and harmful substances may also influence transgenerational wing dimorphism in aphids. For example, precocene, a plant secondary metabolite, stimulates the production of winged offspring in *A. pisum*, which might be attributed to permanent juvenile hormone deficiency (Fridmancohen & Pener, 1980; Hardie, 1986).

Some other factors, such as predation, mutualism, and intraspecific crowding, can stimulate wing production in aphids. Wingless aphid produces a large number of winged offspring under the pressure of predator cues in *Aphis gossypii* and *A. pisum* (Balog, Mehrparvar, & Weisser, 2013; Podjasek, Bosnjak, Brooker, & Mondor, 2005; Weisser, Braendle, & Minoretti, 1999). However, the exposure of *Aphis fabae* and *Megoura viciae* to foraging lacewing (*Chrysoperla carnea*) larvae did not induce winged offspring production (Kunert, Schmoock‐Ortlepp, Reissmann, Creutzburg, & Weisser, 2008). To respond to predator attack, aphids release altruistic alarm pheromone, *E*‐β‐farnesene (EBF), to warn adjacent companions to immediately escape or fall (Sloggett & Weisser, 2002; Weisser et al., 1999). Maternal *A. fabae* aphids tended by the mutualistic ant *Formica fusca* produce a high proportion of wingless offspring, and this phenomenon might be related to a juvenile hormone‐related chemical from the ants (Kleinjan & Mittler, 1975). Moreover, the high‐density signaling increases the proportion of producing winged individuals in *Aphis glycines*, *Megoura crassicauda*, and *A. pisum* (Ishikawa, Gotoh, Abe, & Miura, 2013; Ishikawa & Miura, 2013; Martinez & Costamagna, 2018; Wilkinson et al., 2016). Such maternal stress‐induced wing dimorphism has been a research hotspot for more than a century. However, whether or not these factors modulate the production of wing dimorphism through a common or distinct mechanism remains elusive.

The studies of wing morph differentiation and development have greatly laid a foundation of understanding the condition‐dependent wing dimorphism (wing polyphenism) in viviparous female aphids (Brisson, 2010). Wing differentiation occurred at the early postembryonic instars, that is, at the first to second instar when the wing primordia and flight muscle appeared to develop, and subsequently, the flight muscles were degenerated in the destined wingless nymphs (Grantham & Brisson, 2018; Ishikawa, Hongo, & Miura, 2008). The wingless nymphs might transfer the nutrition and energy from the degraded flight muscle to postembryonic development, suggesting a tradeoff between dispersion and reproduction traits.

Recent breakthrough findings have unveiled some specific molecular mechanisms underlying the regulation of maternal stress‐induced wing dimorphism. Transcriptome analysis of crowding stress‐exposed pea aphids revealed prominent expression changes in genes associated with odorant binding, neurotransmitter transport, hormonal activity, and chromatin remodeling (Vellichirammal, Madayiputhiya, & Brisson, 2016). Specifically, crowding stress repressed the production of three monoamines, namely serotonin, dopamine, and octopamine in brain. The titers of these monoamines might signal developing embryos to be winged or wingless. Another study showed that the number of winged offspring was decreased by RNAi knockdown of the expression of a key octopamine synthesis enzyme, the tyramine β‐hydroxylase (TβH; Wang, Zhang, Zhang, Tian, & Liu, 2016). The result partly confirmed the role of octopamine in the regulation of wing dimorphism in the pea aphids. A subsequent study demonstrated that ecdysone signaling pathway is also involved in the transgenerational wing determination (Vellichirammal, Gupta, Hall, & Brisson, 2017). Females injected with ecdysone or its analog produced more winged offspring. On the contrary, ecdysone signaling suppressed by RNA interference targeting the ecdysone receptor (EcR) or by an EcR antagonist decreased the proportion of winged offspring. Insulin‐related peptide 5 gene (*Apirp5*) regulated the alternation of wing morphs in pea aphids by affecting some physiological phenotypes such as body weight, embryo size, and carbohydrate and protein contents (Guo, Zhang, & Liu, 2016). The insulin signaling pathway could be also involved in the regulation of wing development in pea aphid. In addition, two laterally transferred viral genes, *Apns‐1* and *Apns‐2*, could contribute to the production of winged offspring (Parker & Brisson, 2019). Despite these findings, the molecular mechanism for the regulation of maternal stress‐induced wing dimorphism largely remains unclear.

This study aims to examine the effects of three representative external stimulations, that is, crowding, predation, and alarm pheromone, on the production of winged aphids and to further investigate the molecular mechanisms underlying maternal stress‐induced wing dimorphism. Our findings may advance our understanding on the ecological adaptive mechanism of insect wing dimorphism under stressful environments.

## 2 MATERIALS AND METHODS

### 2.1 Insect

**Figure 1:**
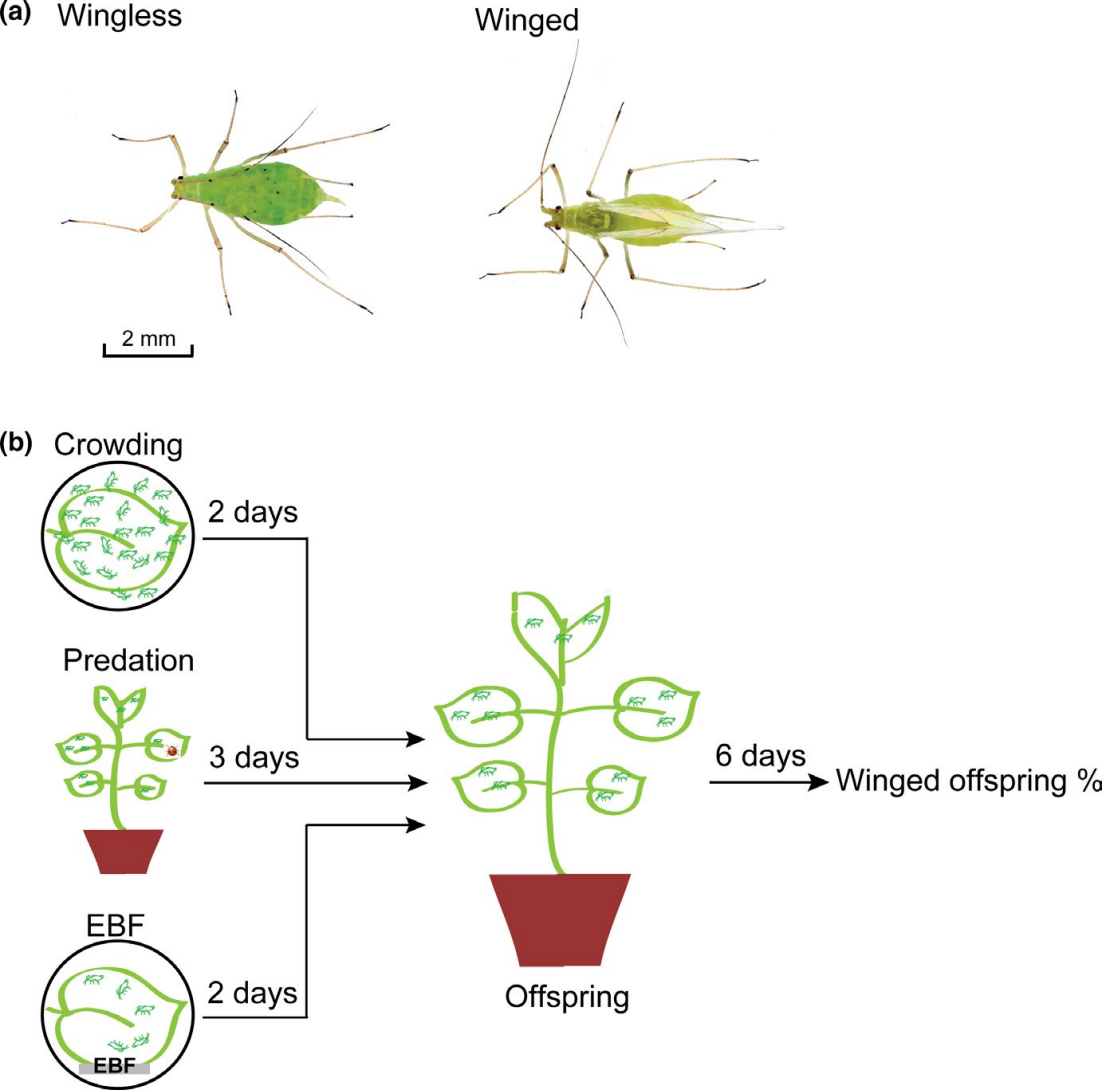
Experimental setup of maternal stress‐induced transgenerational wing dimorphism in pea aphids. (a) Wingless and winged green pea aphids. (b) Experimental design for the induction of aphid wing dimorphism by three maternal stressors, that is, crowding, predation, and alarm pheromone, *E*‐β‐farnesene (EBF). To explore aphid density that caused the most significant crowding effect, we placed females (*n* = 1, 2, 5, 10, 30, or 50) in a Petri dish (8.5 cm) for 2 days, collected the offspring produced on the 2nd day, and counted the number of winged offspring after 6 days. To investigate the induction effect of predation, we placed one ladybird and 30 maternal aphids on a seedling for 3 days and collected all the offspring. No ladybird was placed in the control group. To test the effect of EBF, we placed five aphids in a Petri dish (8.5 cm) under EBF (5 µl, 100 ng/µl) or hexane stimulation five times per day (10:00, 12:00, 14:00, 16:00, and 18:00) for successive 2 days. The number of winged offspring on the 2nd day was counted 6 days later

Green pea aphids (*Acyrthosiphum pisim*; Figure 1a) collected from Yunnan province, China in 2010, were reared on 2‐week‐old broad bean (*Vicia faba*) seedlings (Strain Linchan No. 7, Linxia Seed Company), in an incubator at 23 ± 0.5°C, 70% ± 5% relative humidity, and a 16L:8D photoperiod. The plants were watered daily and fertilized once a week. A parthenogenetic female was randomly selected to start a wingless clone at low density, approximately 20 aphids per seedling, to eliminate cross‐generational effects (Sutherland, 1969a). All maternal aphids used in the experiment were from the same wingless clone and grown to the 8th day until maturity.

Adults of the two‐spot ladybird, *Adalia bipunctata*, were collected from their natural habitats in Dongling Mountain, Beijing, China in July, 2015 and bred in groups of five in 8.5 cm diameter Petri dishes at 25 ± 0.5°C and a 16L:8D photoperiod. The beetles were fed with live pea aphids and readily laid clusters of eggs.

### 2.2 Maternal stress I: Crowding

For this experiment, 1, 2, 5, 10, 30, or 50 adult individuals were placed on a leaf with the petiole wrapped with wet medical absorbent cotton and sealed by parafilm to maintain freshness in a Petri dish (8.5 cm) to manipulate pea aphid maternal density. A layer of filter paper dampened with a few drops of distilled water was placed on the inside bottom of the Petri dish to keep the moisture. Each density treatment was repeated 10 times. After 24 hr, the maternal adults in every treatment were transferred to a new Petri dish with a fresh leaf to continue the density treatment for another 24 hr. The newly born nymphs on the 2nd day were collected and transferred to a new seedling. After 6 days, the offspring reached the 4th instar stage when winged or wingless morphs are easily distinguishable by the presence of wing bud, and the winged offspring were then counted (Figure 1b).

### 2.3 Maternal stress II: Predation

Thirty adult aphids and a two‐spot ladybird adult were placed together on a bean seedling covered with a transparent cylindrical bottle (diameter 7 cm, height 28 cm). The top of the bottle was covered with cotton gauze to prevent the ladybird from escaping. After 3 days, the ladybird and the surviving maternal aphids were removed from the plants (Weisser et al., 1999). In the control group, no presence of any predator was found. All offspring produced in 3 days were left on the bean seedlings until they reached the 4th instar for the determination of the wing form and subsequent calculation of the percentage of winged offspring (Figure 1b). Each treatment was repeated 10 times.

### 2.4 Maternal stress III: Alarm pheromone (EBF)

Five aphids were placed in a Petri dish (8.5 cm) with a fresh leaf that was treated as described above to keep its freshness. For EBF exposure, a filter paper strip (1 × 2 cm), which was added with 5 µl of 100 ng/µl EBF (Sigma‐Aldrich, dissolved in HPLC‐grade hexane) five times per day (10:00, 12:00, 14:00, 16:00, and 18:00) for 2 days, was placed in the Petri dish (Kunert, Otto, Röse, Gershenzon, & Weisser, 2010). The aphids in control groups received 5 µl of hexane at the same time points. The treatment and control Petri dishes were kept in different incubators to ensure that no alarm pheromone affect the control groups. The leaves were replaced daily. All offspring produced on the 2nd day were counted and transferred to fresh bean seedlings until they reached the 4th instar for the determination of the wing form (Figure 1b). Each treatment was repeated 10 times.

### 2.5 RNA extraction and preparation of Illumina sequencing libraries for RNA‐Seq

After 2 days of stress exposure, the parent aphids were sampled and snap frozen in liquid nitrogen and stored at −80°C until RNA preparation. Fifty parent aphids were collected for each of four replicate samples for each treatment (i.e., stress exposure or control). Their offspring were reared on fresh bean seedling until they reached the 4th instar to confirm the efficacy of wing induction. The frozen adult aphids were disrupted and homogenized, and the mixture was oscillated by vortex blending for 2 min. RNA isolation and purification were then immediately performed on each sample using an RNeasy Mini Kit (Qiagen) according to the manufacturer's protocol. The purity of RNA was measured using Nanodrop 2000 (Thermo Scientific), and the integrity of RNA was determined by agarose gel electrophoresis and an Agilent 2100 TapeStation analysis (Aligent).

The mRNA was enriched and purified from the 20 µg total RNA of each sample by poly (T) magnetic beads, and then, the purified mRNA was cut into short clips using fragmentation buffer. The short clips were used as templates and reverse transcribed with the mixture of Superscript II (Invitrogen), Rnase H and DNA polymerase I (Illumina) to synthesize double‐strands cDNA. After purification by QiaQuick PCR Purification Kit (Qiagen), cDNA was ligated with adenine at 3′‐end and Illumina PE adapter. Adaptor‐ligated cDNA was then subjected to PCR amplification. The cDNA library quality was determined by Agilent 2200 Bioanalyzer (Aligent). cDNA libraries were sequenced on the Illumina HiSeq 3000 by “paired‐end” method at RiboBio Co. Ltd. A total of 24 libraries, including four repeats for each treatment, were constructed.

The original image data collected from the Illumina HiSeq 3000 platform were converted into raw sequencing reads. Raw reads were processed in order to obtain clean reads by filtering and removing reads with adaptors, short reads, and low‐quality reads. Then, clean reads were produced and assembled into unigene using Trinity (Grabherr et al., 2011). Tophat2 was adopted to map clean reads to the pea aphid genome (AphidBase Official Gene Set v2, http://www.aphidbase.com/) with parameter as follows: (‐read‐mismatches = 2, ‐read‐gap‐length = 2; Trapnell, Pachter, & Salzberg, 2009). ANNOVAR was used to annotate the aligned transcripts in such an order of priority: exon, splicing region, intron, and intergenic region (Wang, Li, & Hakonarson, 2010). The gene model gff file was downloaded from the pea aphid genome.

The clean data were then transformed into RPKM (Expected number of Reads Per Kilobase of transcript sequence per Millions base pairs sequenced) in order to normalize the relative expression level of the matched unigenes (Mortazavi, Williams, McCue, Schaeffer, & Wold, 2008). Differentially expressed genes were determined by setting a fold change cutoff at least 1.5 and *q* value cutoff 0.05. Replicates with the low pairwise Pearson correlation coefficient (*r* < .7) between each others were excluded for further analysis (Table S1). Then, the average value of relative expression level of all the validated biological replicates in each treatment was used for transcriptome analysis.

### 2.6 Bioinformatics analysis

Enrichment analysis for the supplied gene list was carried out based on an algorithm presented by GOstat (Beissbarth & Speed, 2004), with the whole annotated gene set as the background. The *p*‐value was approximated using the chi‐square test. Fisher's exact test was used when any expected value of count was below 5. Gene Ontology (GO) analysis including three ontologies, “Biological process,” “Cellular component,” and “Molecular function,” provided a standardized gene functional classification method of all DEGs. The WEGO software was used for GO functional classification, and GO terms with a corrected *p*‐value <.05 were defined as significantly enriched GO terms (Audic & Claverie, 1997). Kyoto Encyclopedia of Genes and Genomes (KEGG) analysis was used to identify significantly enriched metabolic pathways or signal transduction pathways in DEGs based on the database at a criteria of *p*‐value < .05 (Kanehisa et al., 2008).

Venn diagrams were constructed on an online website (http://bioinformatics.psb.ugent.be/webtools/Venn/). Heat map was generated using Cluster 3.0 and Treeview (Eisen, Spellman, Brown, & Botstein, 1998). Motifs were identified by MEME (Bailey et al., 2009). Pfam domains of amino acid sequences were predicted via SMART (Letunic, Doerks, & Bork, 2012). Multiple sequence alignment of CP genes was conducted using the MUSCLE program according to the corresponding Pfam domain (Edgar, 2004). Conserved motif analysis of the 23 CP genes shown in the form of Pfam domain logos was performed using Weblogo3 (Crooks, Hon, Chandonia, & Brenner, 2004). Family classification of CP genes was analyzed based on the CuticleDB (http://bioinformatics2.biol.uoa.gr/cuticleDB/index.jsp; Magkrioti, Spyropoulos, Iconomidou, Willis, & Hamodrakas, 2004). Other graphs were plotted with GraphPad Prism5.

### 2.7 Quantitative real‐time PCR (qPCR)

Total RNA was extracted from the antenna, head, gut, cuticle, embryo, and leg using TRIzol reagent (Invitrogen) to test the tissue‐specific distribution of cuticular protein (CP) genes of *A. pisum*. The relative expression of mRNAs was quantified by qPCR utilizing a Light Cycler 480 instrument (Roche) in a 10 µl reaction volume consisting of 5 µl SYBR Green 1 Master Mix (Roche), 1 µl cDNA template, 3 µl H_2_O, and 0.5 µl primer F/R. qPCR procedure was set as follows: 95°C for 2 min, 45 cycles of 95°C for 30 s, 60°C for 30 s, and 72°C for 30 s. Gene‐specific primers were designed with Primer Premier 5 according to the corresponding gene sequences, and unique amplification was verified by melting curve analysis (Table S2). Three biological replicates were assayed for statistical analysis. The 16S gene was selected as the internal control gene (Yang, Pan, Liu, & Zhou, 2014), and the relative expression level was analyzed by 2^−ΔΔCT^ method.

### 2.8 Statistics

Data on the percentages of winged progenies were arcsine square root [*X*′ = arcsin(sqrt(*x*))] transformed and analyzed by SPSS 21.0 software (SPSS Inc). Significant differences between treatments were tested by one‐way ANOVA or *t* test (*p* < .05).

## 3 RESULTS

### 3.1 The three types of maternal stress‐induced prominent wing dimorphism in pea aphids

**Figure 2:**
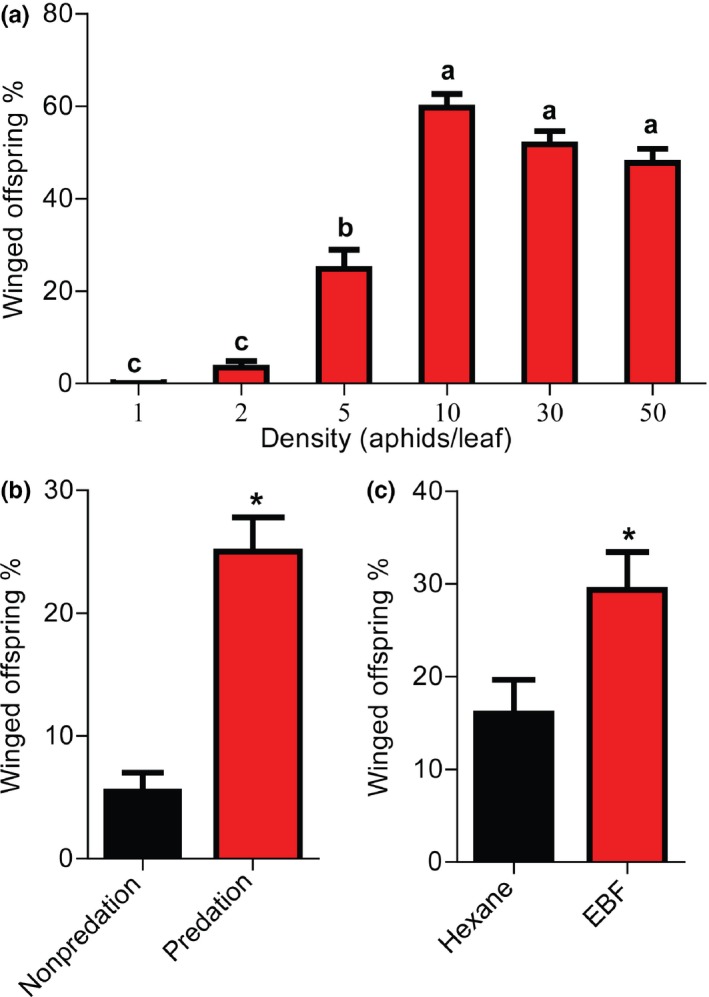
Effect of maternal stressors crowding, predation, and alarm pheromone on offspring wing dimorphism in pea aphids. (a) Effect of maternal density on the proportion of wing dimorphism. Female aphids were crowded at six densities for 2 days. Winged aphids were tallied on the 2nd day of crowding. Different letters indicate significant differences (*p* < .05) using Tukey's multiple range tests. (b) Effect of predation by a ladybird on offspring wing dimorphism. Thirty aphids were cohoused with one ladybird on a leaf for 3 days in each biological replicate. Winged aphids were tallied in the 3 days of treatment. (c) Effect of maternal exposure to alarm pheromone (EBF) on wing dimorphism. Five aphids on a leaf were exposed to 100 ng/µl EBF or hexane. Production of wing morphs was examined on the 2nd day after 2 consecutive days of treatment. Asterisk indicates significant differences between treatment and control (Student's *t* test, *p* < .05). Values in all panels represent mean ± *SE*. Ten biological replicates were used

We investigated whether or not three types of environmental stress, that is, crowding, predation, and alarm pheromone, induced wing dimorphism in pea aphid offspring. We first examined the effect of crowding stress by rearing wingless aphid mothers at different densities before tallying their winged offspring count. Figure 2a depicts the percentage of winged offspring produced by the females on the 2nd day of their crowding treatment. The solitary aphids did not produce any winged offspring. Low density (two aphids/leaf) had no remarkable effect on wing induction, leading to the winged progeny accounting for only 3%. However, the crowding density of five aphids/leaf significantly enhanced the effects (Student's *t* test, *p* < .0001) of this stressor and increased the percentage of winged offspring to 25%. The percentage of winged offspring peaked at 60% for the group density of 10 aphids/leaf (Student's *t* test, *p* < .0001, compared with that of solitary aphids). Further increase in group density of up to 30 and 50 aphids/leaf also induced a high percentage of winged morphs, but the percentage was not higher than that with the density of 10 aphids/leaf. These results indicate that the production of winged aphids can be induced by a certain range of maternal crowding density, and excessive crowding does not enhance the induction effect.

Second, we investigated whether or not the presence of an aphid predator, that is, *A*. *bipunctata*, has a maternal effect on wing dimorphism. Pea aphid adults were subjected to predation stress by rearing 30 adult wingless aphids and one ladybird together on a seedling for 3 days. The percentage of winged offspring was significantly higher in mother aphids with predation exposure (25%) than that in the control without predation exposure (5%; *p* < .0001; Figure 2b).

Finally, the effect of the exposure of mother aphids to alarm pheromone (i.e., EBF) on the production of winged offspring was determined. The aphids reared at a density of five aphids/leaf responded strongly to EBF. The percentage of winged offspring in mother aphids with EBF exposure was significantly higher (29%) compared with that in the hexane control without EBF exposure (16%; *p* < .0001; Figure 2c). These results indicate that EBF stimulates winged morph production in crowded aphids.

### 3.2 Transcriptome analysis reveals divergent molecular responses to the three maternal stress factors

To unveil the molecular mechanisms for the regulation of maternal stress‐induced wing dimorphism, we sequenced the transcriptomes of the stress‐exposed mother aphids that have produced winged morphs and of the nonstressed aphids. We excluded those biological replicates having low correlation of expression among replicates from the same treatment and included four replicates for crowding analysis, three replicates for predation analysis, and two replicates for EBF analysis.

**Figure 3:**
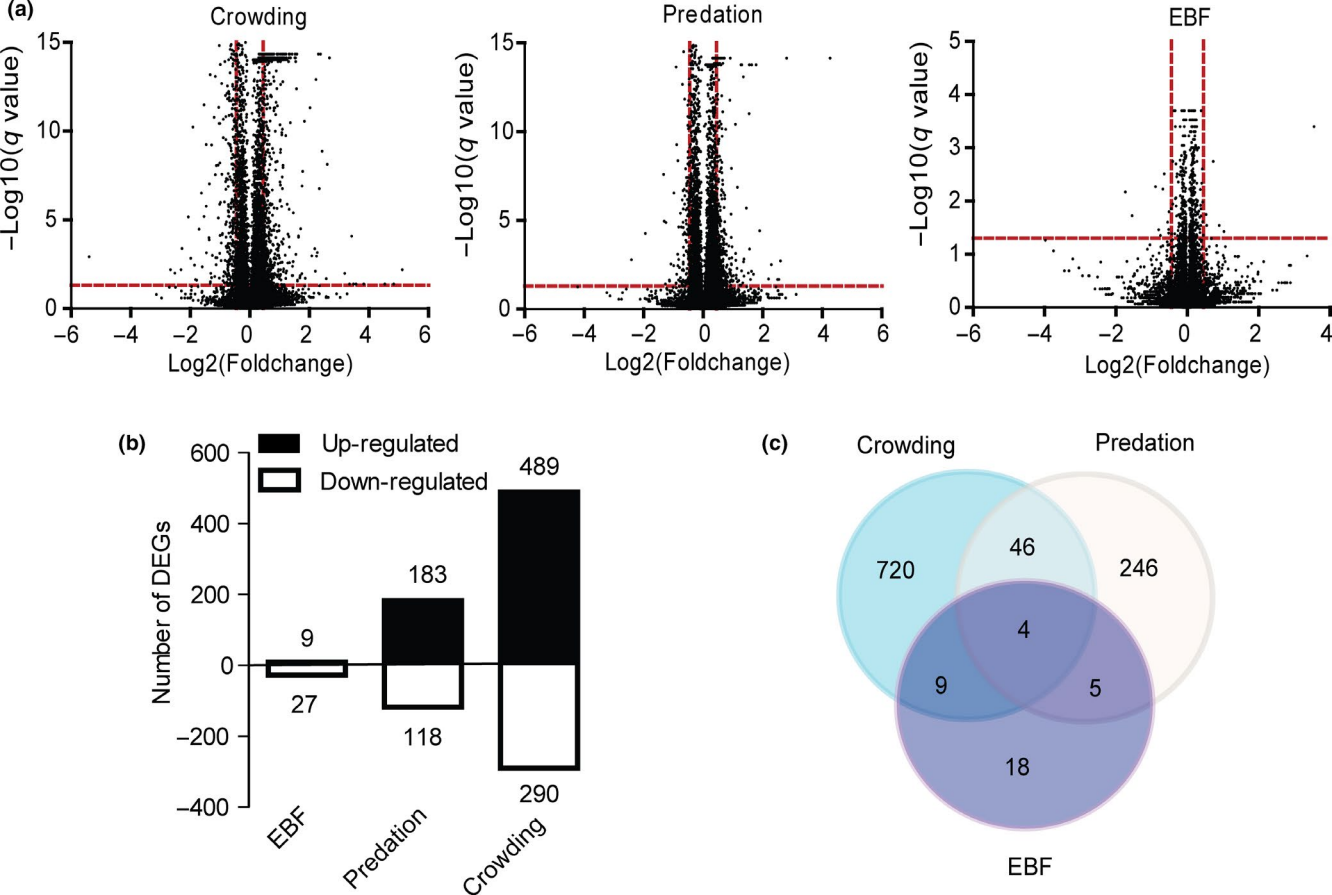
Stressors‐induced maternal transcriptome response in pea aphids. (a) Volcano plot of three stressors‐treated maternal transcriptome. The red dotted lines indicate *q* value = 0.05, |fold change| = 1.5. The dots located in upper left corner of the red dotted lines (*q* value <0.05, fold change < −1.5) represent the significantly downregulated genes; the dots located in upper right corner of the red dotted lines (*q* value < 0.05, fold change > 1.5) represent the significantly upregulated genes. (b) Number of DEGs responding to the three types of maternal stress. DEGs are genes with expression levels altered by stress exposure by fold change >1.5 and *q* value < 0.05. (c) Venn diagram depicting the DEG numbers induced by the three maternal stressors

A total of 12,417, 12,463, and 12,546 expressed transcripts were assembled in crowding, predation, and EBF treatments, respectively (Tables S3–S5 and Figure 3a). Different numbers of genes were significantly elicited by the three stressors, and most of the genes were induced by maternal crowding. A total of 489 genes were upregulated, and 290 genes were downregulated in response to crowding (Figure 3b). In predation treatment, 183 genes were upregulated, and 118 genes were downregulated (Figure 3b).

Only a small number of DEGs (36), including 17 annotated DEGs, were detected in response to the EBF signal (Figure 3b). The majority of these genes presented a moderate (i.e., 1.5 < fold change < 2) but significant (*q* < 0.05) change in their expression level (Table 1). Among the annotated DEGs, only *cp43* and AF4/FMR2 family member 4 were upregulated. Among the downregulated DEGs, *cp45* expression was highly and significantly induced by the EBF signal. Molecular chaperones, including three heat shock protein 68‐like genes and one heat shock protein 70‐like gene, were significantly downregulated. Other downregulated DEGs include titin, microtubule‐associated protein futsch, nesprin‐1, GPI‐anchored adhesion, DNA ligase 1, aromatic‐L‐amino acid decarboxylase, longitudinal lacking protein, transcriptional regulator ATRX homolog, and lachesin (Table 1).

**Table 1:** Seventeen annotated DEGs in EBF‐treated maternal sample

Gene ID	Gene annotation	Up/downregulated	Log_2_(Fold_change)	*q*‐value
*cp45*	Cuticular protein 45 precursor	Down	−0.87342	3.35E−49
LOC100165938	Titin‐like	Down	−0.70743	1.58E−38
LOC100159543	Heat shock protein 68‐like	Down	−0.74898	7.62E−32
*cp43*	Cuticular protein 43 precursor	Up	0.73459	1.27E−13
LOC100575018	Microtubule‐associated protein futsch‐like	Down	−1.42960	2.14E−13
LOC100165351	Nesprin‐1‐like	Down	−1.13233	8.48E−12
LOC100163625	Heat shock protein 68‐like	Down	−0.76829	3.96E−07
LOC103308440	Microtubule‐associated protein futsch‐like	Down	−1.50894	1.40E−06
LOC103308441	Probable GPI‐anchored adhesion‐like	Down	−1.30924	1.44E−05
LOC100162273	AF4/FMR2 family member 4‐like	Up	0.655353	4.85E−05
LOC100160289	Heat shock protein 70 A1‐like	Down	−0.67259	6.33E−05
LOC100570349	Heat shock protein 68‐like	Down	−1.19265	7.14E−05
LOC103308203	DNA ligase 1‐like	Down	−0.65252	3.13E−03
LOC100164582	Aromatic‐L‐amino acid decarboxylase isoform X1	Down	−0.89416	5.41E−03
LOC100165473	Longitudinals lacking protein, isoforms H/M/V‐like	Down	−0.68726	6.01E−03
LOC100569533	Transcriptional regulator ATRX homolog isoform X1	Down	−1.55018	0.019
LOC100571599	Lachesin‐like	Down	−0.80660	0.035

The majority of genes appeared a stressor‐specific expression pattern. Venn diagram shows that 92%, 82%, and 50% of the DEGs in response to crowding, predation, and EBF, respectively, were stress‐specifically expressed (Figure 3c). Only four common DEGs, including a CP gene *cp43* and three unannotated genes, LOC100575566, LOC100574209, and LOC100574390 (Figure 3c), were shared by the three stressors.

**Figure 4:**
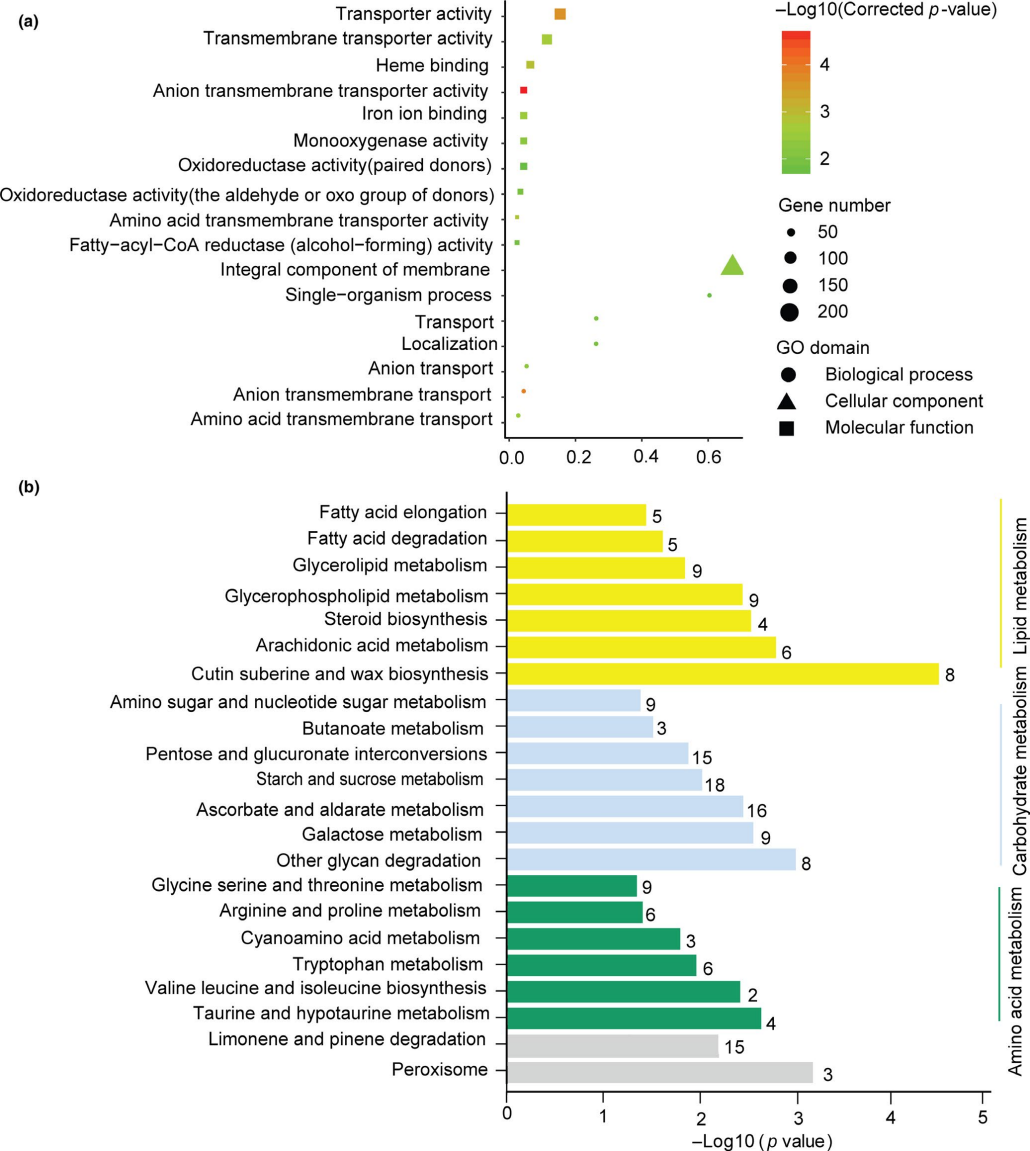
Functional classification of GO and KEGG for DEGs in crowding treatment. (a) GO classification of DEGs in crowding treatment. The *x*‐axis shows rich factor (Cluster frequency), and the *y*‐axis shows significantly enriched GO terms. The color of symbol represents different −log_10_ (Corrected *p*‐value). The size of symbol represents the number of DEGs in each GO term. (b) KEGG classification of DEGs in crowding treatment (*p* < .05). The *y*‐axis indicates the KEGG terms, and the *x*‐axis indicates −log_10_ (*p* value). The number beside each bar indicates the number of DEGs in each KEGG term. The KEGG terms in the same subcategories are presented in same color

We further analyzed the functional involvement of the crowding‐responsive genes and pathways by KEGG analysis. Three main functional categories, that is, lipid metabolism (7 pathways), amino acid metabolism (6 pathways), and carbohydrate metabolism (7 pathways), were significantly enriched (*p* < .05; Table S6). The major pathways involved in lipid metabolism are represented by glycerolipid/glycerphospholipid metabolism and cutin, suberine, and wax biosynthesis (Figure 4b). The subcategories with the largest number of DEGs in amino acid metabolism are responsible for the metabolism of starch, sucrose, ascorbate, and aldarate and the interconversions between pentose and glucuronate. Most of the DEGs involved in carbohydrate metabolism belong to the subcategories that control the metabolism of glycine, serine, threonine, arginine, proline, and tryptophan. These pathways are related to nutrient accumulation and energy mobilization. GO analysis revealed the involvement of transmembrane transport of anion, amino acid, organic anion, and intercellular signal transductions in the crowding‐induced transgenerational wing production (Table S6 and Figure 4a).

### 3.3 Cuticular protein genes were predominantly enriched and highly repressed in response to predation stress

**Figure 5:**
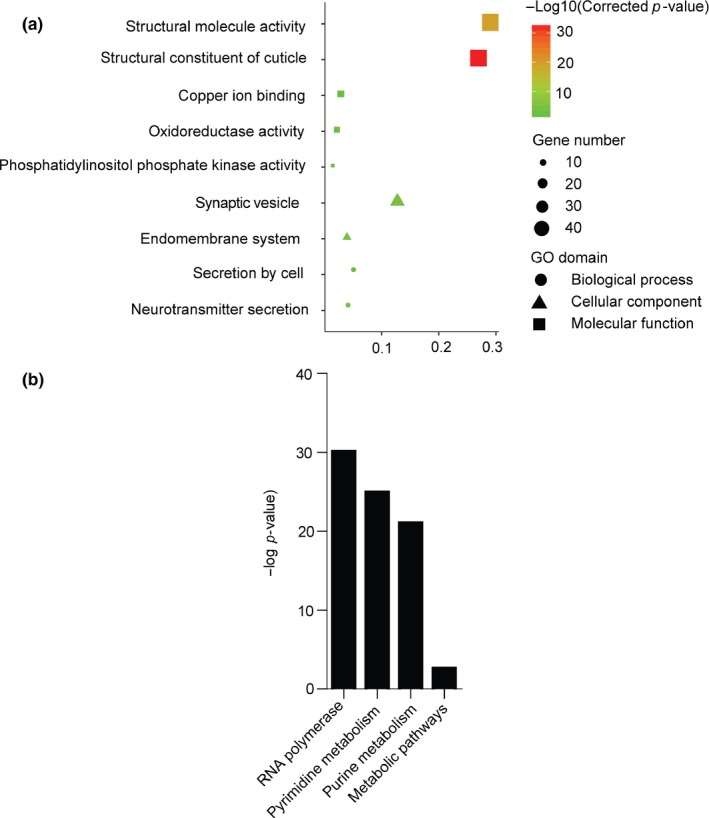
Functional classification of GO and KEGG for DEGs in predation treatment. (a) GO classification of DEGs in predation treatment. The *x*‐axis shows rich factor (Cluster frequency), and the *y*‐axis shows significantly enriched GO terms. The color of symbol represents different −log_10_ (Corrected *p*‐value). The size of symbol represents the number of DEGs in each GO term. (b) KEGG classification of DEGs in predation treatment (*p* < .05). The *y*‐axis indicates the KEGG terms, and the *x*‐axis indicates −log_10_ (*p* value). The number beside each bar indicates the number of DEGs in each KEGG term. The KEGG terms in the same subcategories are presented in same color

We performed GO term analysis of the maternal transcriptomes induced by ladybird predation. In the molecular function domain, the structural molecule activity and structural constituent of cuticle were predominantly enriched (Figure 5a). In addition, genes functioning in neurotransmitter secretion, synaptic vesicle, and cell secretion were also significantly enriched and were closely associated with neuronal signaling and secretion (Table 2, Figure 5a and Table S7). All the 28 annotated genes enriched in these terms belong to CP families (Table 2). KEGG analysis revealed significantly enriched four pathways, in which the annotated DEGs were all CP genes except for *cprr 1‐2* (Table S7 and Figure 5b).

**Table 2:** Gene list of each GO term enriched in predation‐treated maternal sample

GO ID	GO term	DEGs in each term
GO:0008021	Synaptic vesicle	LOC100161786, LOC100164067, LOC100574413, LOC100574328
GO:0012505	Endomembrane system	*cp23*, *cp33*, *cp35*, *cp38*, LOC100161110, LOC100161786, LOC100164067, LOC100168554, LOC100572897, LOC100574328, LOC100574413, LOC100574951, LOC103310513
GO:0042302	Structural constituent of cuticle	*cprr1‐2*, *cp5*, *cp7*, *cp9*, *cp10*, *cp12*, *cp14*, *cp15*, *cp16*, *cp17*, *cp18*, *cp19*, *cp23*, *cp25*, *cp26*, *cp28*, *cp29*, *cp30*, *cp33*, *cp35*, *cp36*, *cp37*, *cp38*, *cp43*, *cp44*, *cp45*, *cp57*, *cp62*, LOC100158777, LOC100159939, LOC100160252, LOC100168571, LOC100302322, LOC100570172, LOC100574951, LOC103310513, LOC103310638, ACYPI009260
GO:0005198	Structural molecule activity	*cprr1‐2*, *cp5*, *cp7*, *cp9*, *cp10*, *cp12*, *cp14*, *cp15*, *cp16*, *cp17*, *cp18*, *cp19*, *cp23*, *cp25*, *cp26*, *cp28*, *cp29*, *cp30*, *cp33*, *cp35*, *cp36*, *cp37*, *cp38*, *cp43*, *cp44*, *cp45*, *cp57*, *cp62*, LOC100158777, LOC100158866, LOC100159528, LOC100159939, LOC100160252, LOC100166333, LOC100168571, LOC100302322, LOC100570172, LOC100574951, LOC103310513, LOC103310638, ACYPI009260
GO:0016715	Oxidoreductase activity	*cp25*, *cp26*, LOC100168571
GO:0005507	Copper ion binding	*cp25*, *cp26*, LOC100168571, LOC100169473
GO:0016307	Phosphatidylinositol phosphate kinase activity	LOC100160785, LOC100165112, LOC100570673, LOC100572974
GO:0007269	Neurotransmitter secretion	LOC100161786, LOC100164067, LOC100574328, LOC100574413
GO:0032940	Secretion by cell	LOC100160317, LOC100161786, LOC100164067, LOC100574328, LOC100574413

**Figure 6:**
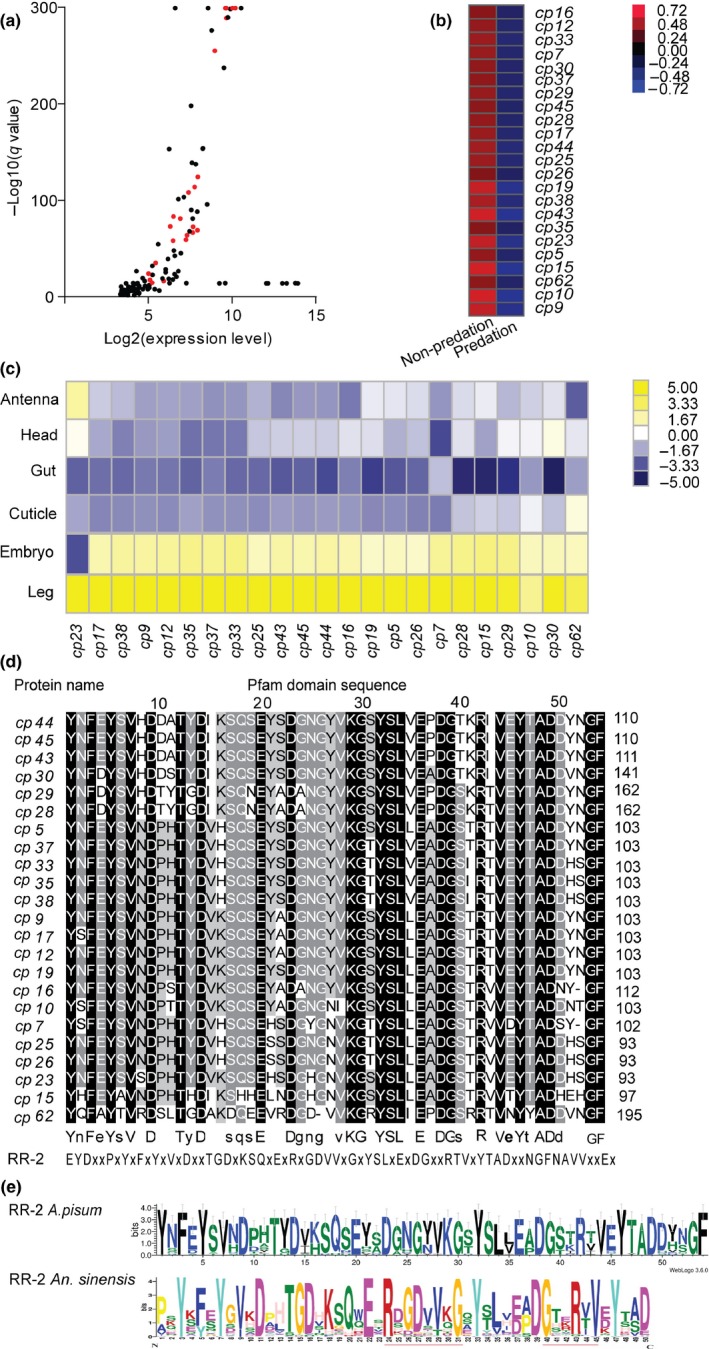
Expression and sequence analysis of 23 cuticular protein (CP) genes regulated by predation stress. (a) Relationship between expression level and *q* value of the top 50 DEGs in predation treatment. The top 50 DEGs were selected based on their *q* values of expression difference from the transcriptome data. The red dots represent the 23 CP genes among the DEGs. (b) Expression levels of the 23 CP genes in the predation treatment based on the transcriptome data. (c) Tissue‐specific expression of the 23 CP genes. Six tissues, that is, antenna, head, gut, cuticle, embryo, and leg, were examined. (d) Pfam domain sequence alignment of 23 CP genes. The 24 highly identical amino acid residues are highlighted in black and partial similarity in gray background. The motif of RR‐2 family provided by cuticleDB is presented. (e) Conserved motifs of RR‐2 subfamily of CP genes in *A. pisum* and *An. sinensis* genome. The 54 aa of Pfam unique motif for 23 RR‐2 genes in *A. pisum* and 50 aa of Pfam unique motif for 102 RR‐2 genes in *An. sinensis*

We also performed a comprehensive analysis on these CP genes in pea aphid. There are 23 CPs among the 50 top DEGs based on their *q* value of expression difference in response to predation. Expression level–*q* value correlation analysis showed that the majority of these CP genes presented high expression level relative to other DEGs (Figure 6a). RNA‐seq data also indicated that the expression levels of the 23 CP genes in mother aphids were all significantly repressed by the presence of the ladybird (Figure 6b).

We further examined the tissue‐specific expression of the 23 CP genes in pea aphids by quantitative PCR (see raw data in Table S8). All the CP genes were highly expressed in the leg tissues. Twenty‐three CP genes, except gene *cp23*, were also highly expressed in embryos (although lower than that in legs; Figure 6c). These CP genes exhibit generally low expression in outer cuticles.

Finally, we also characterized the protein sequences of these CP genes. MUSCLE alignment of the predicted domain sequences indicated that the protein sequences of the 23 CP genes were highly similar, having 24 identical amino acid residues (Figure 6d). Protein family analysis based on the CuticleDB indicated that the 23 CP genes all belonged to RR2 subfamily and all presented a sequence motif that was homologous to the RR‐2 motif reported in *Anopheles sinensis* (Figure 6e; Liu et al., 2017).

## 4 DISCUSSION

This study reported the prominent effects of different maternal stress factors, that is, crowding, predation, and alarm pheromone, on the induction of transgenerational wing dimorphism in aphids. Increasing physical contact of pea aphids in a certain range of density caused many winged progenies (Lees, 1967; Martinez & Costamagna, 2018). Previous studies have revealed that contact between conspecific aphids or stimulation with a paintbrush induces the production of winged offspring (Johnson, 1965; Purandare, Tenhumberg, & Brisson, 2014). Predation and alarm pheromone are strong cues that can drive aphids to walk around and physically encounter each other, consequently resulting in an increased production of the winged progenies. Winged morph reproduction might be the only opportunity to escape from predation for a clone because a predator could result in the extinction of a whole aphid colony (Weisser et al., 1999). The transgenerational effects of maternal stress on wing dimorphism are thus crucial for the survival of aphid colonies in nature.

The transcriptome profiling of maternal crowding stress in this study provides insights into the role of nutrient accumulation and energy mobilization in determining transgenerational wing dimorphism. Functional clustering of DEGs revealed that a considerable number of differentially expressed genes are primarily associated with lipid, amino acid, and carbohydrate metabolism. This result was consistent with previous findings that the parthenogenetic lineages of pea aphid producing many winged individuals exhibited a high metabolic rate (Artacho, Figueroa, Cortes, Simon, & Nespolo, 2011). Such metabolic involvement in crowding response was attributed to a high energy cost to produce winged embryos than wingless ones. The red morph of *A. pisum* produced higher content of lipids and soluble carbohydrates, the only two important storage fuels in *A. pisum*, than the green morph (Ahsaei, Tabadkani, Hosseininaveh, Allahyari, & Bigham, 2013). This form was more sensitive to ecological change and tended to produce more winged offspring under stresses. In addition, the content of carbohydrate and protein in the third‐instar nymphs of the winged morph, whose wing primordia beginning to grow rapidly, is significantly higher than that in the wingless morph (Guo, Jiang, Yi, Liu, & Zhang, 2016). Thus, these two nutrients are vital for the development of primordia during this period. The nutrient accumulation and energy mobilization involved in the regulation of transgenerational wing dimorphism in *A. pisum* may represent a maternal adaptive strategy, in which resources are highly devoted to the development of winged embryos in a crowded population.

The results also imply an active involvement of amino acid catabolism in the regulation of wing dimorphism. In KEGG pathway for valine, leucine, and isoleucine biosynthesis, several genes were upregulated, including a gene encoding threonine dehydratase (LOC100165866), an allosterically controlled enzyme specific for L‐isoleucine synthesis, and branched‐chain‐amino acid transaminase (LOC100167587) that catalyzes the final reaction of leucine, isoleucine, and valine biosynthesis (Umbarger, Neidhardt, & Curtiss, 1996). The gene (LOC100161005) encoding pyrroline‐5‐carboxylate reductase that catalyzes the final step in proline synthesis was also upregulated. By contrast, the gene LOC100161119 encoding ornithine decarboxylase that catalyzes proline into polyamine was downregulated (Wrighton & Busslinger, 1993). These results suggest that proline accumulates with the development of winged embryos. The upregulation of a γ‐glutamyltranspeptidase gene (LOC100165936) in taurine and hypotaurine metabolism promotes amino acid translocation (Sastre, Sweiry, Doolabh, Vina, & Mann, 1991; Smith, Gibson, Howlin, & Pratt, 1991). In fact, amino acids, particularly essential amino acids (EAAs), are vital for insect survival, fecundity, and wing dimorphism (Attardo, Hansen, Shiao, & Raikhel, 2006; Grandison, Piper, & Partridge, 2009; Wilkinson & Ishikawa, 2000). For parthenogenetic aphids, their nutritionally unbalanced phloem sap diet lacks EAAs that are indispensable for rapid embryonic growth (Rabatel et al., 2013). Their EAAs are synthesized from non‐EAA precursors in the phloem sap by obligate *Buchnera aphidicola* (γ‐proteobacterium), which is housed in the bacteriocytes of aphids (Guo et al., 2013; Hansen & Moran, 2011; Shigenobu, Watanabe, Hattori, Sakaki, & Ishikawa, 2000). Consequently, the removal of *Buchnera* with the antibiotic rifampicin substantially decreases the percentage of winged offspring (Zhang et al., 2015). A sharp increase in the percentage of wingless offspring occurs when one EAA (methionine, isoleucine, or histidine) is omitted from the artificial diet used to feed maternal peach aphids. However, omission of non‐EAA (alanine, tryptophane, or yaline) increases the proportion of winged offspring (Awram, 1968; Dadd, 1968; Williams, Dewar, Dixon, & Thornhill, 2000). These studies provide evidence for a close link between amino acid metabolism and transgenerational wing dimorphism in *A. pisum*.

The transcriptome analysis of maternal crowding also suggests that carbohydrates are required for the development of winged embryos. The composition and concentration of carbohydrates can influence the growth and reproduction of aphids. For example, sucrose (concentration 10%–30%) is a dominating sugar in the phloem sap of plants and the preferred and irreplaceable sugar in the artificial diet for *A. pisum* (Auclair, 1965; Mitchell, Smale, & Metcalf, 1960). Larviposition and survival rates are low when 13% trehalose without sucrose was supplemented in their diet. Galactose‐supplemented diet significantly restrains wing production (Raccah, Tahori, & Applebaum, 1972). In the galactose metabolism pathway, galactokinase (LOC100165228) and galactose‐1‐phosphate uridylyltransferase (LOC100168691), which were upregulated at high levels, are crucial in catalyzing galactose to the metabolically useful glucose‐6‐phosphate. In addition, the crowding‐induced glycogen phosphorylase (LOC100159778) and hexokinase (LOC100169524) can catalyze the conversion of glucose to glucose‐6‐phosphate (G6P; Patra & Hay, 2013). We speculated that G6P might partially account for the accumulation of energy reserves for the muscular activity of locomotion and the response to crowding stress.

Expression analysis of predation response revealed a widespread involvement of CP genes in the regulation of wing dimorphism in pea aphids. Twenty‐three CP genes and cuticle‐associated GO terms were significantly enriched in the predation‐induced treatment. A recent comparative proteomics study reported that 100 members belonging to five CP families (RR‐1, RR‐2, CPAP1, CPF, and TWDL) existed in the cast cuticles of pea aphids, with 72 members belonging to RR‐2 family (Masson, Arafah, Voisin, & Bulet, 2018). CP genes are involved in the synthesis of epidermis and nonstructural components, such as pigments, enzymes, defense proteins, and arylphorin (Kornezos & Chia, 1992; Leung, Palli, & Locke, 1989; Marcu & Locke, 1998; Molnar, Borhegyi, Csikos, & Sass, 2001). Among the five families, the RR‐1 family in exocuticle and RR‐2 family in endocuticle can bind to chitin (Bouligand model) to regulate cuticle flexibility and rigidity (Andersen, 1998, 2000; Moussian, 2010). Knockdown of 14 different CP genes in brown planthopper causes the endocuticles to be thin and disordered (Pan et al., 2018). One CP gene is downregulated in maternal nutrition stress‐induced transgenerational wing dimorphism in *A. pisum* (Jedlicka, Jedlickova, & Lee, 2015). CP genes have also been found to be involved in sexual size dimorphism in the ghost moth *Thitarodes pui* (Guo, Jiang, et al., 2016). These studies revealed the diverse regulatory roles of CP genes in insects, although many are not yet appreciated. Our study suggested that CP genes could regulate transgenerational wing dimorphism via sensing and transduction of external environment stimuli such as predator presence. For pseudoplacental viviparity aphids, the eggshell of oocytes and embryos without yolk and chorion might facilitate immediate and flexible transgenerational transmission of various environmental information from mother aphid to its embryos (Ogawa & Miura, 2014). The unique localization of some CP genes in cuticle facilitates the response of internal tissues to external environment. Almost all of these CP genes are highly expressed in leg tissues (Figure 6c). Arthropods benefit to a large extent from a jointed cuticular exoskeleton that senses multiple environmental stimuli by mechanoreceptors, chemical receptors, and vibration detectors on their legs (Delcomyn, Nelson, & Cocatre‐Zilgien, 1996). *Cp23* is highly expressed in antenna receiving olfactory stimuli. Amputation of the antennae in pea aphid under crowding stress substantially reduces the percentage of winged offspring (Sutherland, 1969a). In addition, the high expression level of all CP genes in embryo suggests their active involvement in embryonic development and wing morph determination. However, the contribution of these CP genes in regulating wing development in embryo or transducing environmental signal to the embryos remains unknown.

In conclusion, our study provides a comprehensive understanding of the molecular genetic basis for the determination of winged descendants. The expression of CP genes related to transgenerational wing morphs can be induced by predation, crowding, and EBF stresses. The results imply a common regulatory mechanism underlying the transgenerational effects of these three stressful factors. However, stress factor‐specific mechanisms are widespread in the regulation of wing form dimorphism. The responses to various stressors enable pea aphids to cope with environment changes and ensure their population persistence in growing seasons. Our comparative analyses have profound implications in understanding the divergent evolution of the regulation of phenotypic plasticity under distinct selection pressures.

## CONFLICT OF INTEREST

None declared.

## AUTHOR CONTRIBUTIONS

Li Chen and Bing Chen conceived the study, designed the experiments, analyzed the data, and revised the manuscript. Lin Hu performed the experiments on wing dimorphism induction and RNA sample collection. Wanying Gui analyzed the transcriptome data, performed qPCR, and wrote the manuscript.

## Data Availability

The transcriptome data were deposited in NCBI SRA: PRJNA507326. The file “Supplementary Table” was deposited in Dryad (https://doi.org/10.5061/dryad.55b2b15).
